# OTUD1 ameliorates cerebral ischemic injury through inhibiting inflammation by disrupting K63-linked deubiquitination of RIP2

**DOI:** 10.1186/s12974-023-02968-7

**Published:** 2023-11-27

**Authors:** Shengnan Zheng, Yiquan Li, Xiaomeng Song, Mengting Wu, Lu Yu, Gan Huang, Tengfei Liu, Lei Zhang, Mingmei Shang, Qingfen Zhu, Chengjiang Gao, Lin Chen, Huiqing Liu

**Affiliations:** 1 Department of Pharmacology, School of Basic Medical Sciences, Shandong University, Jinan, Shandong 250012 People’s Republic of China; 2 Key Laboratory of Infection and Immunity of Shandong Province & Department of Immunology, School of Basic Medical Sciences, Shandong University, Jinan, Shandong 250012 People’s Republic of China; 3 Rheumatology Unit, Department of Medicine, Solna, Karolinska Institute, Karolinska University Hospital, Stockholm, Sweden; 4 Shandong Institute for Food and Drug Control, Jinan, Shandong 250012 People’s Republic of China; 5 Department of Rehabilitation Medicine, The Second Hospital, Cheeloo College of Medicine, Shandong University, Jinan, Shandong 250012 People’s Republic of China

**Keywords:** Cerebral ischemic injury, RIP2, OTUD1, Inflammation, Ubiquitination

## Abstract

**Background:**

Inflammatory response triggered by innate immunity plays a pivotal element in the progress of ischemic stroke. Receptor-interacting kinase 2 (RIP2) is implicated in maintaining immunity homeostasis and regulating inflammatory response. However, the underlying mechanism of RIP2 in ischemic stroke is still not well understood. Hence, the study investigated the role and the ubiquitination regulatory mechanism of RIP2 in ischemic stroke.

**Methods:**

Focal cerebral ischemia was introduced by middle cerebral artery occlusion (MCAO) in wild-type (WT) and OTUD1-deficient (OTUD1^−/−^) mice, oxygen glucose deprivation and reoxygenation (OGD/R) models in BV2 cells and primary cultured astrocytes were performed for monitoring of experimental stroke. GSK2983559 (GSK559), a RIP2 inhibitor was intraventricularly administered 30 min before MCAO. Mice brain tissues were collected for TTC staining and histopathology. Protein expression of RIP2, OTUD1, p-NF-κB–p65 and IκBα was determined by western blot. Localization of RIP2 and OTUD1 was examined by immunofluorescence. The change of IL-1β, IL-6 and TNF-α was detected by ELISA assay and quantitative real-time polymerase chain reaction. Immunoprecipitation and confocal microscopy were used to study the interaction of RIP2 and OTUD1. The activity of NF-κB was examined by dual-luciferase assay.

**Results:**

Our results showed upregulated protein levels of RIP2 and OTUD1 in microglia and astrocytes in mice subjected to focal cerebral ischemia. Inhibition of RIP2 by GSK559 ameliorated the cerebral ischemic outcome by repressing the NF-κB activity and the inflammatory response. Mechanistically, OTUD1 interacted with RIP2 and sequentially removed the K63-linked polyubiquitin chains of RIP2, thereby inhibiting NF-κB activation. Furthermore, OTUD1 deficiency exacerbated cerebral ischemic injury in response to inflammation induced by RIP2 ubiquitination.

**Conclusions:**

These findings suggested that RIP2 mediated cerebral ischemic lesion via stimulating inflammatory response, and OTUD1 ameliorated brain injury after ischemia through inhibiting RIP2-induced NF-κB activation by specifically cleaving K63-linked ubiquitination of RIP2.

**Supplementary Information:**

The online version contains supplementary material available at 10.1186/s12974-023-02968-7.

## Introduction

Stroke is the most common ground of severe disability and the second leading cause of death in adult worldwide with high global burden [1, 2]. Ischemic stroke caused by cerebral blood flow interruption accounts for approximately 62.4% of all stroke cases and leads to nutrition deficiency and consequently cell death and function loss in brain [3, 4]. In clinical practice, thrombolytic drugs such as rtPA and endovascular thrombectomy are the major treatments for acute ischemic stroke patients. Yet, the benefits of these therapies are limited due to the potential adverse effects and narrow therapeutic time window [5, 6]. Thus, the persistent search for novel therapeutic intervention to improve the ischemic stroke outcomes is necessary.

Brain ischemic lesion causes neurons and glia cells to generate damage-associated molecular patterns (DAMPs), leading to the activation of astrocyte and microglia, consequently producing pro-inflammatory cytokines and chemokines, which trigger inflammatory responses. Our previous study demonstrated that the nucleotide-binding oligomerization domain (NOD) 2 participated in cerebral ischemia in a mouse model by promoting inflammatory response. Moreover, the molecular mechanism of inflammatory damage in ischemic stroke mediated by NOD2 targeted NOX2-dependent oxidative stress [7]. Receptor-interacting kinase (RIPK or RIP) 2 is the key component of NOD2 signaling complex, and its ubiquitination is conducive to the NF-kB inflammatory signaling pathway [8]. Once activated, NOD2 recruits RIP2, which is ubiquitinated by the linear ubiquitin chain assembly complex (LUBAC) and inhibitor of apoptosis proteins (IAP). Meanwhile, deubiquitinating enzymes (DUBs) participate in the disassembling of Met1 and K63-linked ubiquitin chains on RIP2 to restrict signal transduction [9–12]. Therefore, ubiquitination of RIP2 is a critical post-translational modification regulated by ubiquitinases and DUBs. No doubt, regulation of ubiquitination for RIP2 plays indispensible role in NOD2-mediated downstream signaling transduction. However, the mechanism underlying the ubiquitination regulation of RIP2 in cerebral ischemia has not been studied.

DUBs are a series of ubiquitin hydrolases that trim or remove mono- or chained-ubiquitin from substrates [13]. As one of the six distinct DUB families, the ovarian tumor protease (OTU) family modulates inflammation, metabolism and DNA replication signaling pathways [14]. OTUD1 is a member of OTU domain family and previous studies confirmed OTUD1 cleaved ubiquitin chains of transcription factors and regulators, such as p53 [15], YAP [16] and IRF3 [17]. Our previous study showed OTUD1 played an essential role in antifungal innate immunity by deubiquitinating Caspase recruitment domain-containing protein 9 (CARD9) and regulating the CARD9 signaling pathway [18]. In addition, OTUD1 reduced interferon production induced by RIG-I-like receptors [17]. In cancer development OTUD1 served as a tumor suppressor and participated in innate immunity. Whether OTUD1 influences the ubiquitination of RIP2 and regulates the inflammatory response in cerebral ischemia remains unclear.

In this study, we demonstrated that RIP2 mediated cerebral ischemic lesion via promoting inflammatory response both in vivo and in vitro*.* In addition, OTUD1 ameliorated brain injury after ischemia through inhibiting RIP2-induced inflammation by cleaving its K63-linked ubiquitination.

## Materials and methods

### Mice

C57BL/6 wild-type (WT) (male, 8–10 W, 23 ± 3 g, animal approval number: SCXK (Lu) 2019-0001) mice were purchased from Animal Center of Shandong University. OTUD1^−/−^ mice obtained from Professor Chengjiang Gao (Shandong University, Jinan, China) were described previously [18]. All experimental procedures were approved by the Ethics Committee of Shandong University and in accordance with the Institutional Animal Care and Use Committee of Shandong University.

### Regents and plasmids

GSK2983559 (TopScience, Cat. T5401) was purchased from TopScience. Poly-d-lysine hydrobromide (Sigma, Cat. P0899) and 2, 3, 5-Triphenyltetrazolium chloride (TTC) (Sigma, Cat. T8877) were obtained from Sigma.

The vectors encoding OTUD1 (WT and C320A) and ubiquitin (WT, K63 and K48), the NF-κB firefly luciferase reporter plasmid and pGL3 promoter-dependent Renilla luciferase reporter plasmid was kindly provided by Professor Chengjiang Gao [18]. His-RIP2 plasmid (Human, GV219) was purchased from GENE.

### Primers and siRNAs

The sequences of the primers used in qRT-PCR were listed in Table 1. The 3 siRNAs targeting OTUD1 were designed as below:

**Table 1:** Sequences of the primers

Genes of interest	Primers
β-actin	5ʹ-AGGGCTATGCTCTCCCTCAC-3ʹ 5ʹ-CTCTCAGCTGTGGT GGTGAA-3ʹ
IL-6	5ʹ-ACAACCACGGCCTTCCCTAC-3ʹ 5ʹ-CATTTCCACGATTTCCCAGA-3ʹ
IL-1β	5ʹ-AACCTCTTCGAGGCACAAGG-3ʹ 5ʹ-GGCGAGCTCAGGTACTTCTG-3ʹ
TNF-α	5ʹ-GTGAAGGGAATGGGTGTT-3ʹ 5ʹ-GGTCACTGTCCCAGCATC-3ʹ

1st: 5-CCACUUCAGCCCACUCAUUTT-3;

2nd: 5-CCGGAUAUCCCGAAUUGCUTT-3;

3rd: 5-GCUCAGCAAUG GACAUAUTT-3.

### Animal model of focal cerebral ischemia

OTUD1^−/−^ and WT mice were subjected to middle cerebral artery occlusion (MCAO) as described previously [7, 19]. Briefly, the mice were initially anesthetized by intraperitoneal injection of 50 mg pentobarbital per kilogram of body weight. The left common carotid artery and left external carotid artery were exposed through a midline neck incision. A MCAO monofilament (CINONTECH, Beijing, Cat. A1-162050) was inserted into the left external carotid artery and advanced into the left internal carotid artery past the MCA origin until the tip reached the proximal anterior cerebral artery, thus occluding the origin of the MCA. A successful occlusion was indicated by a severe reduction in the regional cerebral blood flow to < 20% of the baseline by a laser-Doppler flow meter (Moor, England). Body temperature was kept at 37 °C during operation with a homeothermic blanket. Mice were euthanized after subjected to MCAO without reperfusion at the indicated time. The sham animals were subjected to the same procedure except for the occlusion of the MCA. GSK2983559 (GSK559, RIP2 inhibitor, 3 μl of 200 μM for each mouse) was intraventricularly injected (coordinates: 0.04 mm posterior to the bregma, 0.1 mm lateral to the midline, 0.22 mm ventral to the dura) to mice 30 min before MCAO.

### Cell culture and treatment

BV2 cells were purchased from the China center for type culture collection (Wuhan, China), HEK293T cell was a kind gift from Professor Chengjiang Gao. BV2 cells and HEK293T cells were maintained in Dulbecco’s Modified Eagle’s Medium (DMEM, Meilunbio, Cat. MA0212) supplemented with 10% FBS (LONSERA, Cat. S711-001S) and incubated with 5% CO_2_ at 37 °C.

Primary neuron cells were cultured as described in literature [19]. Briefly, cerebral cortex was isolated from E18 mice and neurons were seeded on six-well plates at the density of 7 × 10^5^/well in B27-containing media (Neurobasal medium, Thermo, Cat. A3582901). Neurons were used for experiments after 7 days of culture.

Primary astrocytes cultures were prepared from postnatal 1–2 day mouse pups as previously described [20]. The cerebral cortex was isolated, minced, treated with trypsin at 37 °C for 30 min. After stopping digestion by DMEM/F12 (Meilunbio, Cat. MA0124) medium containing 10% FBS, cells were passed through 100 μm pore-sized membrane (Merck, Darmstadt, Germany) and seeded in T75 flasks. Culture media were refreshed every 2–3 days. Astrocytes were used for experiments after 7 days of culture.

### Transfection and treatment

Cells were transfected with plasmid or siRNA using Lipofectamine 2000 (Invitrogen, Cat. 11668019). Cells were deprived of oxygen and glucose (OGD) by incubating in EBSS solution for 90 min with 5% CO_2_ and 95% N_2_ at 37 °C. Then, the cells were switched back to reoxygenation (OGD/R) for the indicated time [19].

### Immunofluorescence staining

Frozen brain sections (10 μm) or the slides of cells were double labeled with indicated primary antibodies at 4 °C overnight, then gave the secondary antibodies and incubated for 2 h at room temperature. DAPI (Beyotime, Cat.C1005) was incubated for nuclear staining [19].

Antibodies and dilution ratio used in immunofluorescence staining were as follow: anti-OTUD1 (Abcam, Cat. 122481, Rabbit, 1:100), anti-RIP2 (Proteintech, Cat. 15366-1-AP, Rabbit, 1:100; Santa Cruz, Cat. sc-136059, Mouse, 1:100), anti-Flag (Sigma, Cat. F1804, Mouse, 1:200), anti-6*His (Proteintech, Cat. 66005–1-lg, Mouse, 1:200), anti-NeuN (Millipore, Cat. MAB377, Mouse, 1:200), anti-Iba1 (Proteintech, Cat. 10904–1-AP, Rabbit, 1:100; Santa Cruz, Cat. sc-32725, Mouse, 1:100), anti-GFAP (Millipore, Cat. MAB360, 1:100), Alexa Fluor 488 Goat anti-rabbit IgG Ab (Proteintech, Cat. SA00006-2, 1:200), Alexa Fluor 594 Goat anti-Mouse IgG Ab (Proteintech, Cat. SA00006-3, 1:200).

### TTC staining

After MCAO, mice brains were cut coronally into 2 mm slices. The slices were stained with 0.2% TTC (dissolved in PBS) at 37 ℃ for 30 min and kept in 4% paraformaldehyde (PFA, Sigma, Cat. P6148). Infarct volumes were calculated as: (contralateral volume − healthy ipsilateral volume)/contralateral volume.

### Neurological deficit score in mice

A 4-tiered neurological scoring system were used to determine the outcome by a blinded observer as described previously [21, 22]. Neurological deficit was assessed as follows: 0, normal function; 1, flexion of the torso and contralateral forelimb on lifting the animal by the tail; 2, circling to the contralateral side but normal posture at rest; 3, reclination to the contralateral side at rest; 4, absence of spontaneous motor activity. 5, death.

### Histopathology

Mice brains were collected and fixed in 4% PFA, embedded in paraffin, and sectioned. The sections were stained with hematoxylin and eosin (H&E) (Solarbio, Cat. G1120) to observe the neuronal morphology in the cerebral cortex and hippocampus. TUNEL staining was performed by an in situ cell death detection kit (Roche, Cat. 42134700) according to the manufacturer’s instructions as previously described [23].

### Western blot and immunoprecipitation(IP)

Western blot analysis of the target proteins in cells and brain tissues was performed as previously described [24].The lysates from cells or brain tissues were extracted using RIPA buffer (Beyotime, Cat. P0013B). Protein concentrations were determined using a BCA protein assay reagent kit (Meilunbio, Cat. MA0082) and 40 μg proteins were electrophoresed on SDS–PAGE, then transferred to polyvinyldifluoridine membranes (Millipore, Cat. 3010040001). The membranes were incubated first with the primary antibodies at 4 °C overnight, and then followed by incubation with HRP-conjugated secondary antibodies at room temperature for 2 h.

For immunoprecipitation, cells were lysed with NP40 buffer and centrifuged, first incubated with the indicated antibody and corresponding IgG controls at 4 °C for 3 h, next, mixed with Protein A+G agarose (Santa Cruz, Cat. Sc-2003), after incubated overnight at 4 °C under rotation, the beads were washed and eluted in SDS sample buffer for further analysis.

Antibodies and dilution ratio used in Western blot and IP assay as follow: anti-OTUD1 (Abcam, Cat. 122481, Rabbit, 1:1000), anti-RIP2 (Proteintech, Cat. 15366-1-AP, Rabbit, 1:100; Santa Cruz, Cat. sc-136059, Mouse, 1:1000), anti-Flag (Sigma, Cat. F1804, Mouse, 1:3000), anti-HA (OriGene, Cat. TA180128, Mouse, 1:3000), anti-6*His (Proteintech, Cat. 66005-1-lg, Mouse, 1:3000), anti-IκBα (CST, Cat. 4814, Mouse, 1:1000), anti-p-NF-κB-p65 (CST, Cat. 3033, Rabbit, 1:1000), anti-β-ACTIN (Affinity, Cat. AF7018, 1:5000), anti-GAPDH (Proteintech, Cat. 60004-1-Ig, Rabbit, 1:5000), HRP-conjugated Affinipure Goat Anti-Mouse IgG (H+L) (Proteintech, Cat.SA00001-1, 1:10,000), HRP-conjugated Affinipure Goat Anti-Rabbit IgG (H+L) (Proteintech, Cat.SA00001-2, 1:10,000).

### ELISA assay

The protein levels of IL-6 and TNFα in the supernatants of BV2 and astrocytes cultures were measured by the ELISA kits (eBioscience, E-HSEL-M0003 & E-HSEL-M0009) following the manufacturer’s instructions. Data were quantitatively normalized to the protein concentration of control group.

### Reverse transcription and qPCR

Total RNA was extracted using RNA-Quick purification kit (ES Science, Cat. RN001) and 1 μg RNA was reverse-transcripted into cDNA using Hiscript II Q RT SuperMix for qPCR (Vazyme, Cat. R223-01) with the following procedure: 50 °C, 15 min; 80 °C, 15 s. The qPCR was performed with UltraSYBR One Step RT-qPCR Kit (CWBio, Cat. CW2624) as follow protocol: 45 °C, 10 min; 95 °C, 10 s and 65 °C, 45 s for 40 cycles; 95 °C, 15 s; 60 °C, 1 min; 95 °C, 15 s; 60 °C, 15 s. The result was captured and analyzed with BIORAD IQ5 software.

### Dual-luciferase reporter assay

Plasmids encoding RIP2, OTUD1 (WT or C320A) were co-transfected with NF-κB firefly luciferase reporter plasmid and pGL3 promoter-dependent Renilla luciferase reporter plasmid into HEK293T cells for 36 h by Lipofectamine 2000. Firefly & Renilla Luciferase Reporter Assay Kit (Meilunbio, Cat. MA0518) was used for Luciferase activity.

### Statistical analysis

All results were presented as means ± SD and analyzed with GraphPad Prism 8.0 software. *T* test, one-way or two-way analysis of variance (ANOVA) followed by Tukey’s multiple comparisons test determined the statistical differences between different groups. *P* < 0.05 indicates statistically significant.

## Results

### The protein level of RIP2 was upregulated in WT mice brain subjected to MCAO

**Fig. 1:**
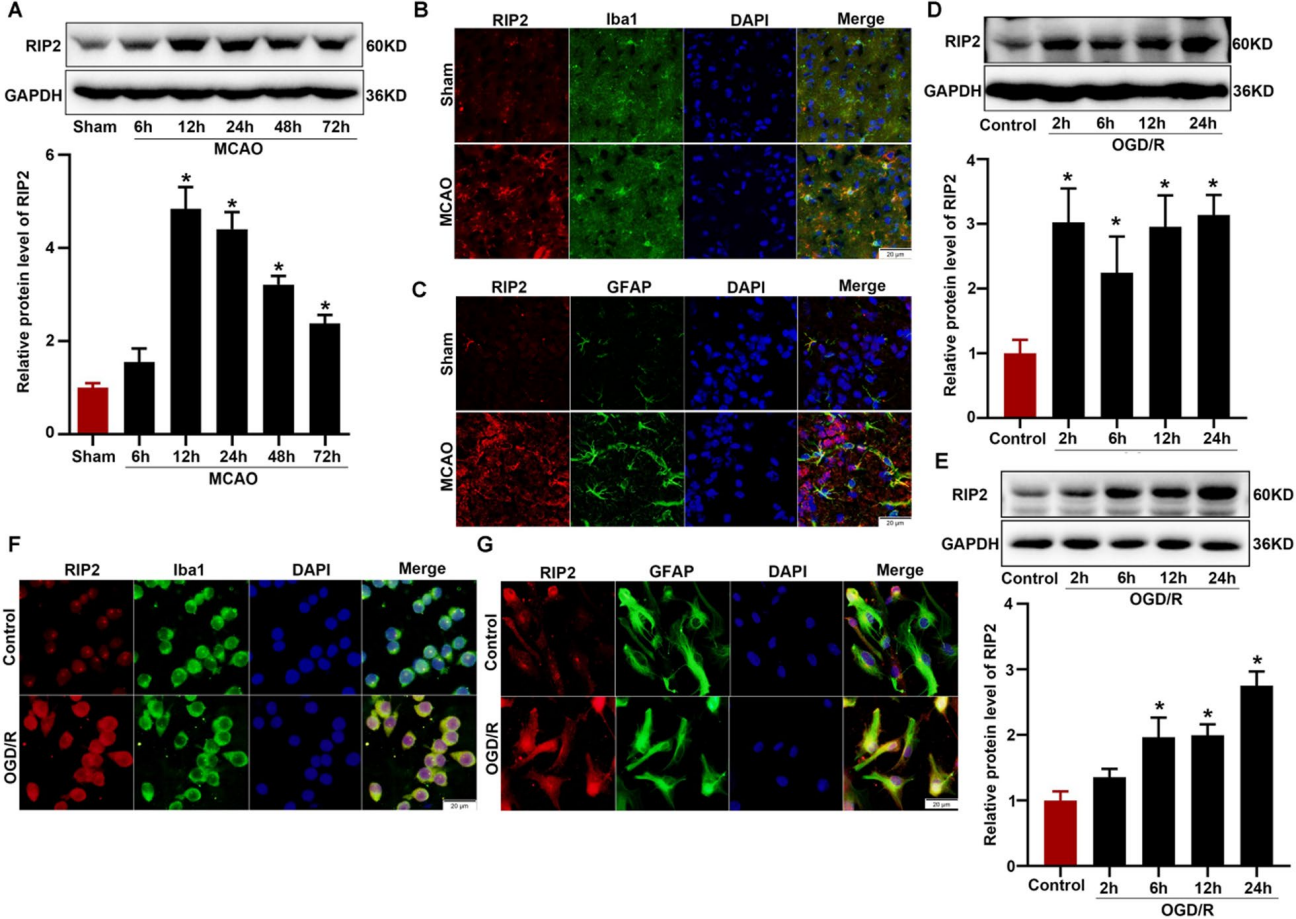
Expression of RIP2 was up-regulated after cerebral ischemia. **A** Western blot of RIP2 expression in ischemic brain tissue from WT mice after 6 h, 12 h, 24 h, 48 h and 72 h middle cerebral artery occlusion (MCAO). *n* = 6 mice per group, one-way ANOVA with Tukey’s multiple comparisons test. *P < 0.05 compared with sham group. **B**, **C** Immunofluorescence of RIP2 in mouse ischemic cortex after 24 h MCAO. Double immunofluorescence of RIP2 (red) and Iba1 (microglial cell marker, green) (**B**), GFAP (astrocyte marker, green) (**C**) were performed. Scale bars: 20 μm. **D** Western blot of RIP2 in BV2 cells subjected to 90 min OGD and 2 h, 6 h, 12 h, 24 h reoxygenation. Results are representative of three independent experiments, one-way ANOVA with Tukey’s multiple comparisons test. *P < 0.05 compared with control group. **E** Western blot of RIP2 in primary cultured astrocytes cells subjected to 90 min OGD and 2 h, 6 h, 12 h, 24 h reoxygenation. Results are representative of three independent experiments, one-way ANOVA with Tukey’s multiple comparisons test. *P < 0.05 compared with control group. **F**, **G** Representative immunofluorescence image of RIP2 in BV2 cells and primary cultured astrocytes. Double immunofluorescence of RIP2 (red) and Iba1 (microglial cell marker, green) (**F**), GFAP (astrocyte marker, green) (**G**) were performed. Scale bars: 20 μm

RIP2 is a pivotal regulator of inflammatory responses to bacterial infections, where it is activated by the NOD1 and NOD2 [25]. To investigate the underlying role of RIP2 in cerebral ischemic injury, we first checked the protein expression of RIP2 in mouse brains triggered by MCAO. We found the RIP2 protein expression was markedly increased in ischemic brain tissue 12 h, 24 h, 48 h and 72 h post MCAO in WT mice (Fig. 1A). We next explored the cell location of RIP2 by double immunofluorescence staining of RIP2 with microglia-specific marker Iba1 and astrocyte-specific marker GFAP. We observed that RIP2 positive cells were co-localized with both Iba1-positive microglia and GFAP-positive astrocytes (Fig. 1B, C). In vitro experiments revealed that RIP2 protein expression was significantly elevated in BV2 cells (Fig. 1D, F) and primary astrocytes (Fig. 1E, G) subjected to OGD/R. These results suggested RIP2 was participated in ischemic stroke and associated with inflammation.

### Inhibition of RIP2 by GSK559 ameliorated the cerebral ischemic outcome

**Fig. 2:**
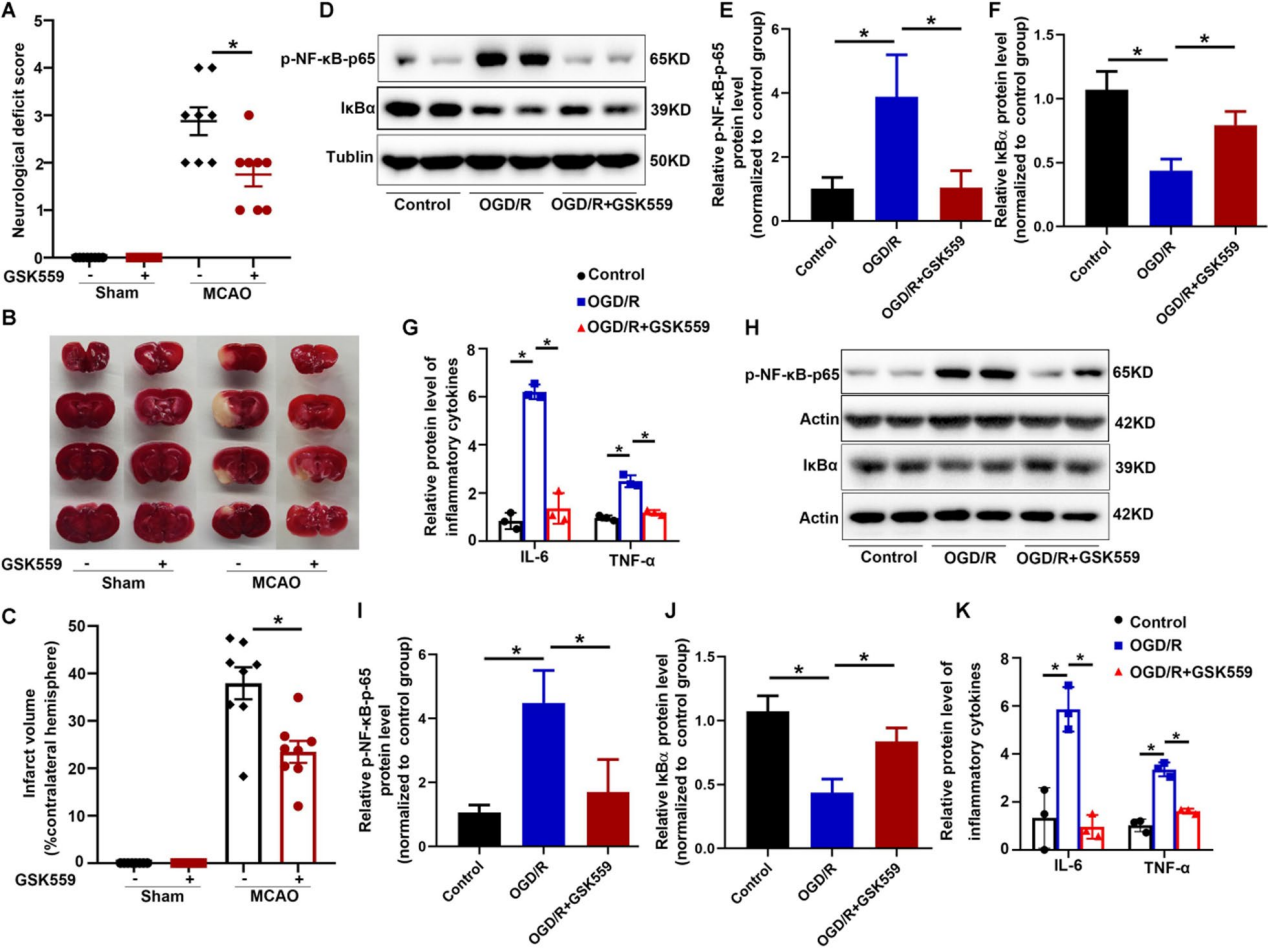
GSK2983559 improved stroke outcomes after cerebral ischemia. WT mice were subjected to 24 h MCAO. GSK2983559, an inhibitor of RIP2 was intraventricularly administered to mice 30 min before MCAO. **A** Neurological deficit scores. *n* = 8 mice per group, two-way ANOVA with Tukey’s multiple comparisons test. *P < 0.05 compared with indicated group. **B** Representative photographs of coronal brain sections following infarction, stained with 2, 3, 5-triphenyltetrazolium chloride (TTC). **C** Summary of cerebral infarct volume in brains. The infarct volume was expressed as the percentage of the contralateral hemispheric area. *n* = 8 mice per group, two-way ANOVA with Tukey’s multiple comparisons test. *P < 0.05 compared with indicated group. Western blot analysis of Phospho-NF-κB p65 and IκBα protein levels in BV2 cells (**D**–**F**) and primary cultured astrocytes (**H**–**J**) after OGD/R. ELISA analysis of proinflammatory cytokine (IL-6 and TNF-a) production in supernatant of BV2 cells (**G**) and primary cultured astrocytes subjected to OGD/R (**K**). Results are representative of three independent experiments, one-way ANOVA with Tukey’s multiple comparisons test. *P < 0.05 compared with indicated group

Using RIP2 inhibitors to ameliorate NOD-mediated pathologies by inhibiting ubiquitination of RIP2 is a potential strategy [26]. We first examined whether RIP2 inhibitor GSK559 has effects on cerebral ischemic injury. Pretreatment with GSK559 attenuated neurological deficit score (Fig. 2A) and reduced infarction volume (Fig. 2B, C) in mice subjected to MCAO. Next, we explored the effect of GSK559 in BV2 cells and primary astrocytes stimulated by OGD/R. Clearly, the increased phosphorylation of p65 subunit of NF-κB and the decreased IκBα protein level indicated the activation of NF-κB pathway upon OGD/R in both BV2 cells (Fig. 2D–F) and primary astrocytes (Fig. 2H–J). More interestingly we observed a marvelous suppression of the NF-κB activation when GSK559 supplied to both cell types (Fig. 2D–F and Fig. 2H–J). Furthermore, the secretion of IL-6 and TNF-α were greatly induced in primary astrocytes and BV2 cells subjected to OGD/R, while GSK559 treatment diminished the induction of these two proinflammatory cytokines (Fig. 2G, K). Collectively, our data indicated that, inhibition of RIP2 protected the brain ischemia by repressing the inflammatory response.

### Upregulation of OTUD1 was triggered by brain ischemia both in vivo and in vitro

**Fig. 3:**
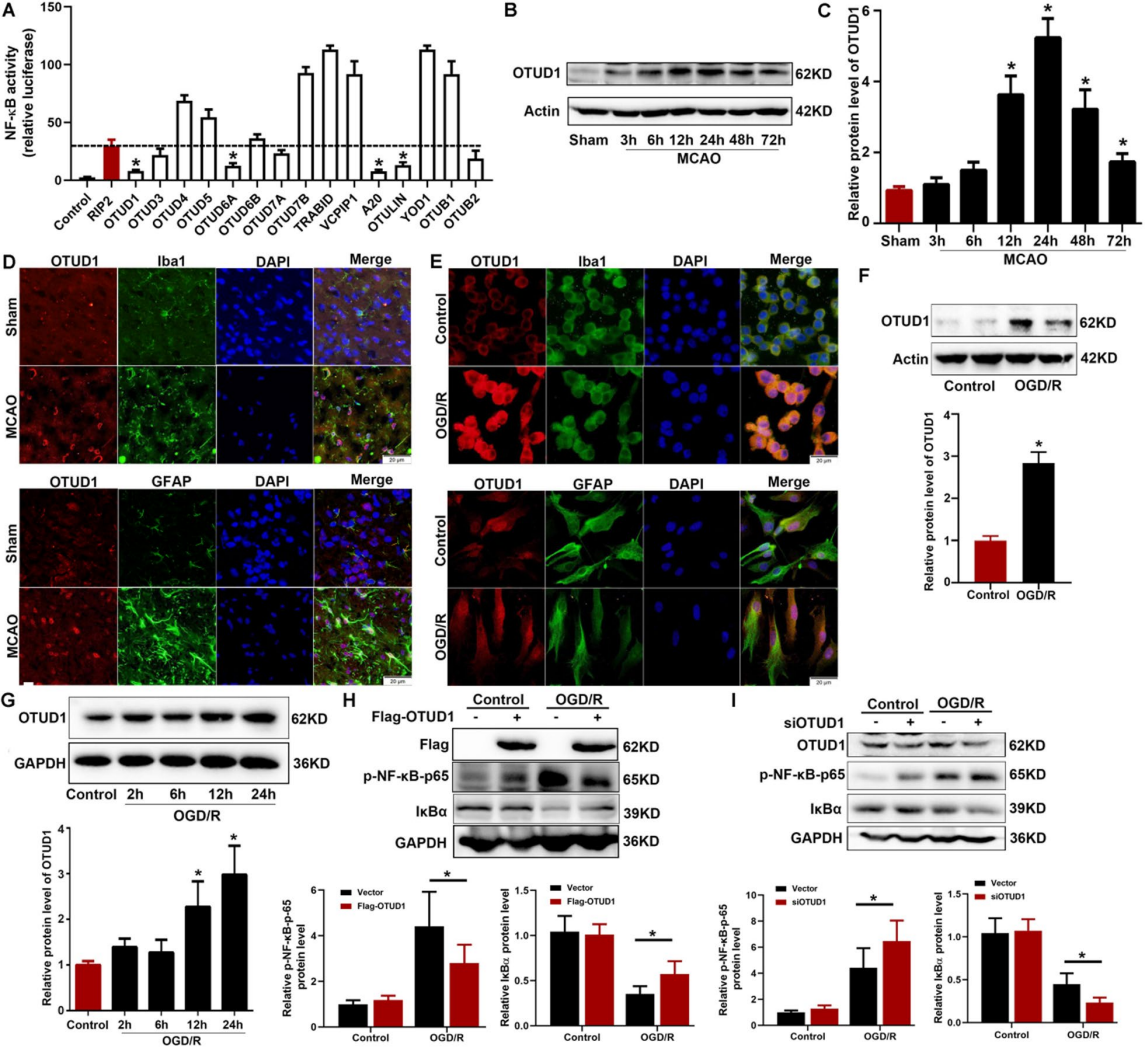
OTUD1 was upregulated and participated in inflammatory response after cerebral ischemia. **A** Luciferase assay of NF-κB activity was used to determine the effect of 15 OUT family members on RIP2-induced NF-κB activity in HEK293 cells. Results are representative of three independent experiments, one-way ANOVA with Tukey’s multiple comparisons test. *P < 0.05 compared with RIP2 group. **B**, **C** Western blot of OTUD1 protein expression in ischemic brain tissue from WT mice after 3 h, 6 h, 12 h, 24 h, 48 h and 72 h MCAO. *n* = 6 mice per group, one-way ANOVA with Tukey’s multiple comparisons test. *P < 0.05 compared with sham group. **D** Representative immunofluorescence image of OTUD1 in mouse ischemic cortex after 24 h MCAO. Double immunofluorescence of RIP2 (red) and Iba1 (microglial cell marker, green) or GFAP (astrocyte marker, green) were performed. Scale bars: 20 μm. **E** Representative immunofluorescence image of OTUD1 in BV2 cells and primary cultured astrocytes subjected to 90 min OGD and 24 h reoxygenation (OGD/R). Scale bars: 20 μm. **F** Western blot of OTUD1 in BV2 cells subjected to OGD/R. Results are representative of three independent experiments. two-tailed unpaired *t* test, *P < 0.05 compared with control group. **G** Western blot analysis of OTUD1 in primary cultured astrocytes cells subjected to 90 min OGD and 2 h, 6 h, 12 h, 24 h reoxygenation. Results are representative of three independent experiments, one-way ANOVA with Tukey’s multiple comparisons test. *P < 0.05 compared with control group. **H** BV2 cells were transfected with either OTUD1 overexpression Flag-OTUD1 vector or control vector for 24 h. Western blot was used to analyse for the protein levels of Flag, p-NF-κB p65 and IκBα in BV2 cells after OGD/R. Results are representative of three independent experiments, two-way ANOVA with Tukey’s multiple comparisons test, *P < 0.05 compared with indicated group. **I** Scrambled siRNA or OTUD1 targeting siRNA were transfected into the BV2 cells. Western blot was used to analyse for the protein levels of OTUD1, p-NF-κB p65 and IκBα in BV2 cells after OGD/R. Results are representative of three independent experiments, two-way ANOVA with Tukey’s multiple comparisons test, *P < 0.05 compared with indicated group

As widely accepted, RIP2 mediated NF-κB activation is tightly regulated by the ubiquitin system, especially some DUBs had been reported as negative regulators [12, 27]. Therefore, we screened for OTU family by luciferase assay for NF-κB activity responding to RIP2 stimulation. The screening results showed that RIP2 induced NF-κB activity was significantly reduced by OTUD1 and A20 compared to other DUBs (Fig. 3A). We investigated whether OTUD1 is involved in cerebral ischemic lesion through modulating inflammation. First, the protein expression of OTUD1 was upregulated in ischemic brain tissue of WT mice after MCAO (Fig. 3B, C), as well as in BV2 cells (Fig. 3F) and primary astrocytes (Fig. 3G) subjected to OGD/R. Next, immunofluorescent analysis showed that OTUD1 was co-localized with markers of microglia and astrocytes both in vivo and in vitro (Fig. 3D, E). Furthermore, OTUD1 overexpression dampened the activation of NF-κB in BV2 cells subjected to OGD/R, while knockdown OTUD1 by siRNA OTUD1 showed increased activity of NF-κB compared with control group after OGD/R stimulation (Fig. 3H, I). Hence, these data indicated that OTUD1 participated in cerebral ischemia as a negatively regulator of NF-κB activation induced by OGD/R.

### OTUD1 interacted with RIP2

**Fig. 4:**
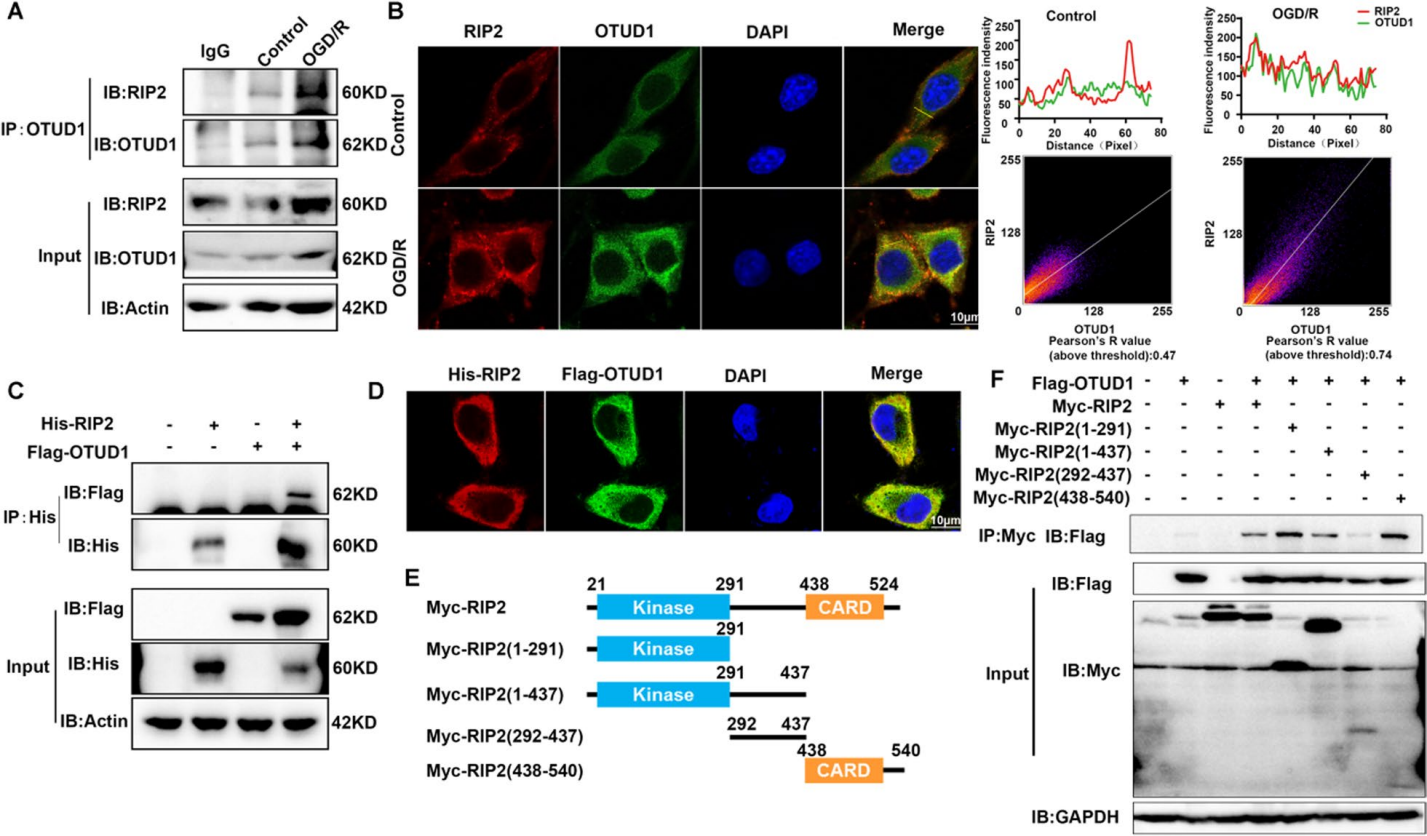
OTUD1 interacted with RIP2. **A** IP analysis of the endogenous interaction of OTUD1 with RIP2 in BV2 cells after the treatment of 90 min OGD and 24 h reoxygenation (OGD/R). **B** Representative images of laser scanning confocal microscopy for RIP2 (red) and OTUD1 (green) in BV2 cells stimulated by OGD/R. Scale bars: 10 μm. **C** IP analysis of the exogenous interaction of OTUD1 with RIP2. HEK293T cells were transfected with plasmids expressing FLAG–OTUD1 and His-RIP2. **D** Representative images of laser scanning confocal microscopy for His (red) and Flag (green) in HEK293T cells. Scale bars: 10 μm. **E** Schematic diagram of RIP2 and its truncation mutants. **F** Myc-tagged RIP2 or its mutants and Flag-OTUD1 were individually transfected into HEK293T cells. The cell lysates were immunoprecipitated with an anti-Myc antibody and then immunoblotted with the indicated antibodies

Given the OTUD1 and RIP2 protein expression were both elevated and localized in microglia and astrocytes of mice with cerebral ischemia, we hypothesize that OTUD1 interacted with RIP2, thus regulate the ubiquitination of RIP2. The endogenous interaction between OTUD1 and RIP2 was determined by Co-IP and immunofluorescence in BV2 cells subjected to OGD/R injury. Compared to the control group, the affinity of OTUD1 and RIP2 was markedly enhanced following the stimulation of OGD/R with increased protein expression of OTUD1 and RIP2 (Fig. 4A). The result was consistent with confocal microscopy, both appreciated the co-localization of OTUD1 and RIP2 (Fig. 4B). Furthermore, His-RIP2 and Flag-OTUD1 were transfected into HEK293T cells, Co-IP experiment and confocal microscopy observed the interaction and colocalization of His-RIP2 with Flag-OTUD1 (Fig. 4C, D). To dig out the necessary domains of RIP2 interaction with OTUD1, a series of truncated mutants of RIP2 were transfected for Co-IP experiments. We found OTUD1 was coprecipitated with RIP2 WT, 1–291, 1–437, 438–540 domain deletion mutant (Fig. 4E, F). Collectively, our data suggested OTUD1 interacted with the kinase domain or CARD domain of RIP2 and may influence the post-translational modification of RIP2.

### OTUD1 removed K63-linked polyubiquitin chains of RIP2

**Fig. 5:**
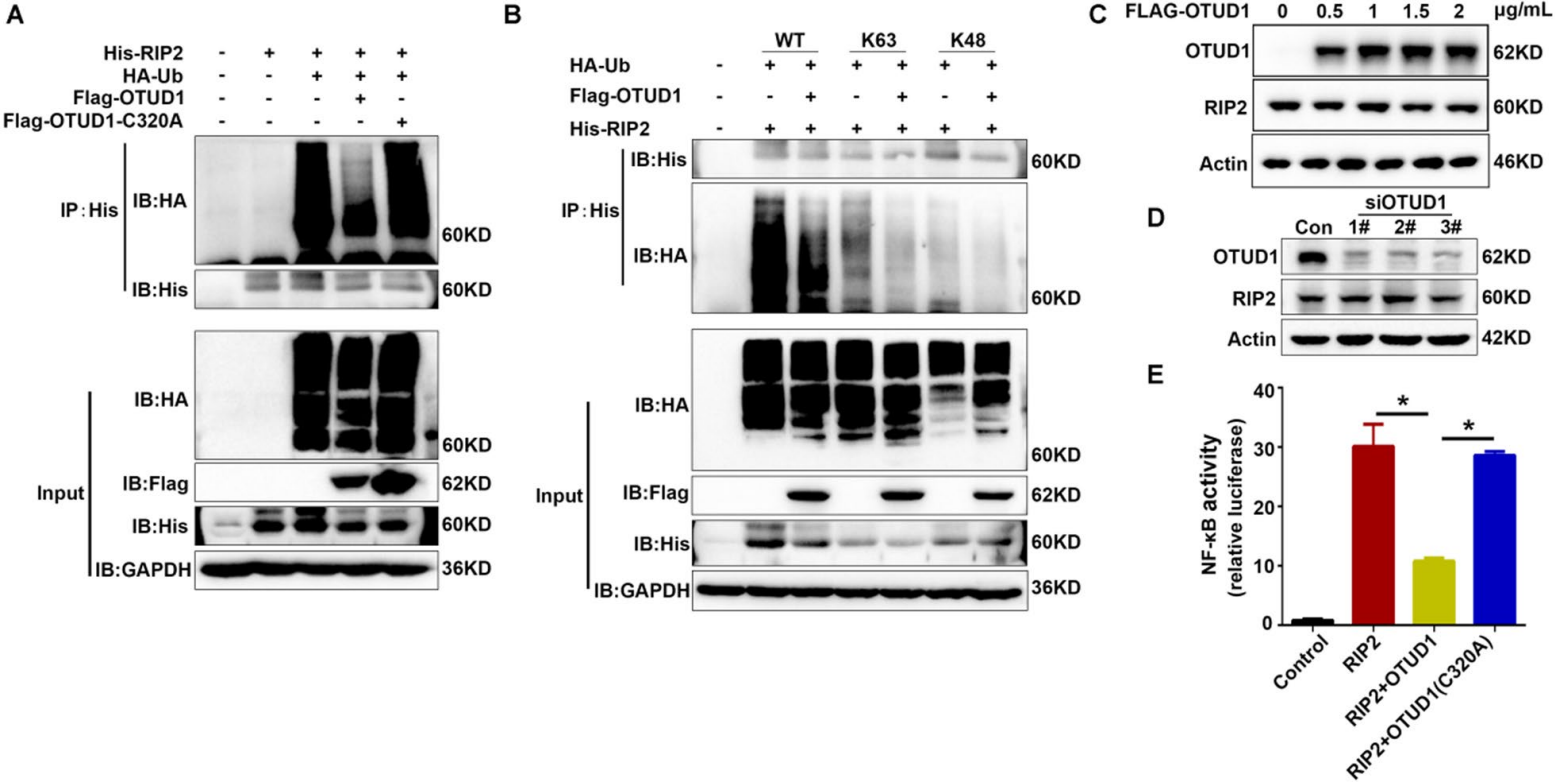
OTUD1 removed K63-linked polyubiquitin chains of RIP2. **A** IP analysis of the deubiquitination of RIP2 transfected with plasmids expressing His-RIP2, HA-ubiquitin (HA-Ub) and Flag-OTUD1 (WT or C320A) in HEK293T cells. **B** HEK293T cells were transfected with plasmids expressing HA-Ub (WT or K48, K63), Flag-OTUD1, and His-RIP2. IP analysis of the polyubiquitination forms of RIP2 regulated by OTUD1. **C** Western blot analysis of RIP2 protein levels after OTUD1 overexpression in HEK 293T cells transfected with plasmids expressing Flag-OTUD1. **D** Western blot analysis of RIP2 in HEK 293T cells transfected with siRNA OTUD1. **E** Luciferase assay of NF-κB activity in HEK293T cells after transfection with control, His-RIP2 or Flag-OTUD1 (WT or C320A) vector together with the NF-κB reporter vector and Renilla luciferase vector for 36 h. Results are representative of three independent experiments, one-way ANOVA with Tukey’s multiple comparisons test, *P < 0.05 compared with indicated group

Next, we assessed whether OTUD1 could regulate the ubiquitination of RIP2. His-tagged RIP2, HA-tagged ubiquitin and Flag-tagged OTUD1 or OTUD1 mutant C320A were co-transfected into HEK293T cells. The ubiquitination of RIP2 was significantly decreased in the co-transfected WT OTUD1 plasmid group (Fig. 5A). Whereas, the enzymatic activity mutant C320A of OTDU1 did not harbor any change on the ubiquitination of RIP2, which suggested that deubiquitination effect of OTUD1 on RIP2 depended on its enzyme activity (Fig. 5A). Next, we assessed which type of ubiquitin chain of RIP2 was cleaved by OTUD1. As shown in Fig. 5B, OTUD1 mainly removed K63-linked but not K48-linked polyubiquitin chains of RIP2. In addition, we studied if OTUD1 regulated the protein level of RIP2. Neither overexpression of OTUD1 by transfected Flag-OTUD1 nor knockdown of OTUD1 by siRNA in HEK293T cells showed any significant change in protein level of RIP2, indicating OTUD1 did not affect RIP2 protein degradation (Fig. 5C, D). Similar results were obtained in BV2 cells (Additional file 1: Figure S1). Meanwhile, luciferase assay confirmed the overexpression of OTUD1 suppressed RIP2-induced NF-κB activation, while OTUD1 (C320A) mutant has no effect on that (Fig. 5E). The above results demonstrated that OTUD1 removed the K63-linked polyubiquitin chains of RIP2 by its enzyme activity and inhibited RIP2-induced NF-κB activation but not affecting the degradation of the RIP2.

### OTUD1 deficiency exacerbated cerebral ischemic injury in response to inflammation induced by RIP2 ubiquitination

**Fig. 6:**
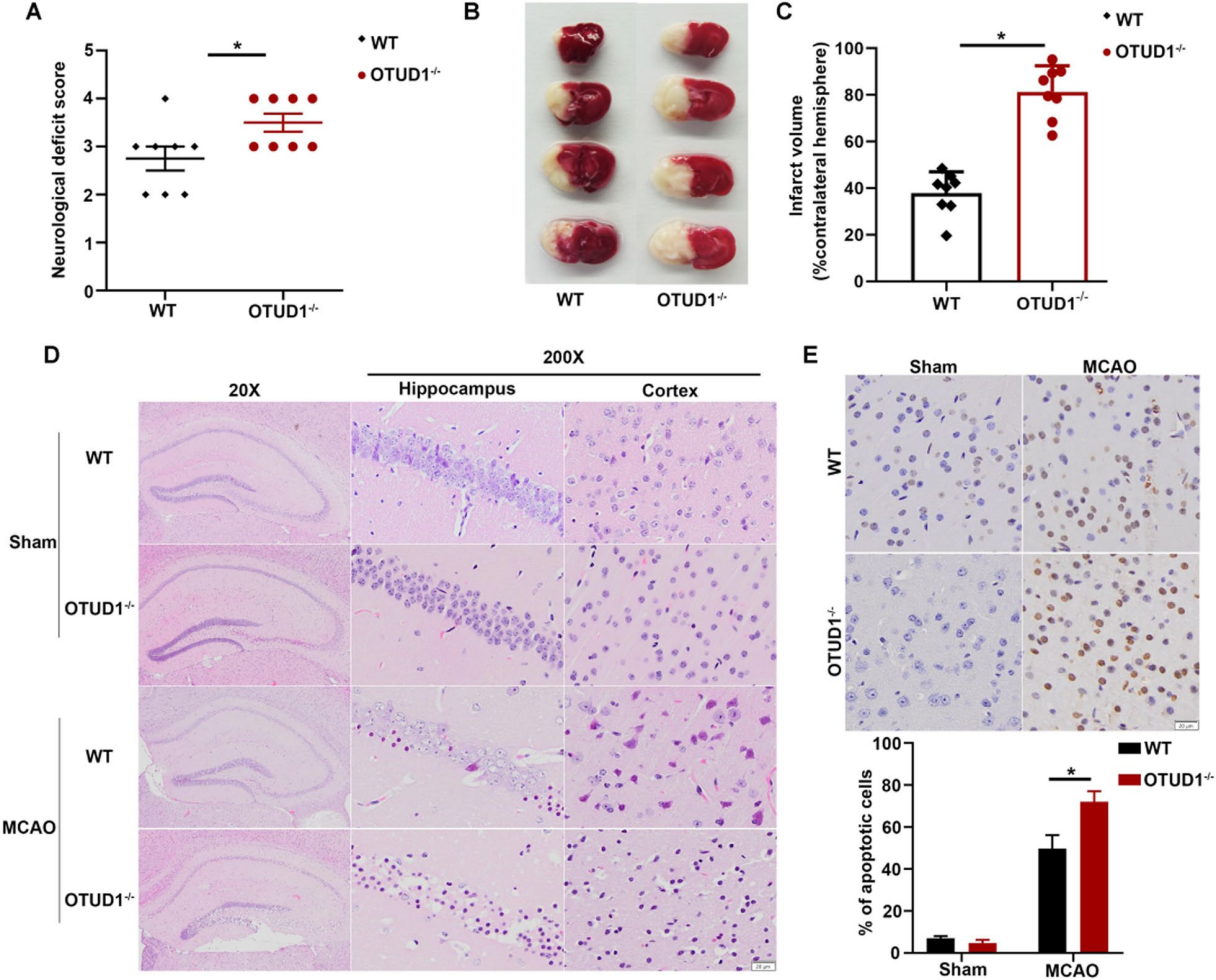
OTUD1 alleviated cerebral ischemic injury in mice. WT and OTUD1^−/−^mice were subjected to the MCAO for 24 h. **A** Neurological deficit score. *n* = 8 mice per group, two-tailed unpaired *t* test, *P < 0.05 compared with MCAO group of WT mice. **B** Representative photographs of coronal brain sections following infarction, stained with 2, 3, 5-triphenyltetrazolium chloride (TTC). **C** Summary of cerebral infarct volume in brains. The infarct volume was expressed as the percentage of the contralateral hemispheric area. *n* = 8 mice per group, two-tailed unpaired *t* test, *P < 0.05 compared with MCAO group of WT mice. **D** Representative photomicrographs of H&E staining in the cortex and hippocampus. Scale bars: 20 μm. **E** Representative images of apoptosis based on TUNEL assay in brain tissue. Scale bars: 20 μm. *n* = 4 mice per group, two-way ANOVA with Tukey’s multiple comparisons test, *P < 0.05 compared with indicated group

**Fig. 7:**
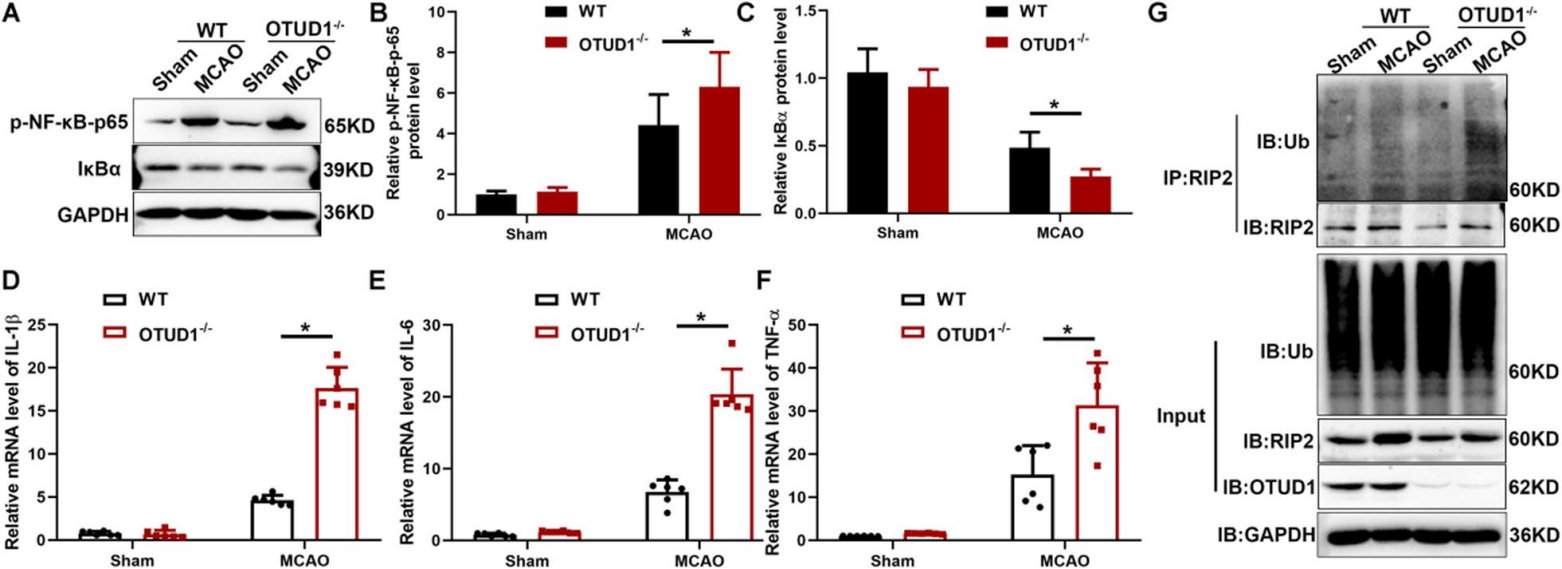
OTUD1 suppressed the inflammatory response by decreasing the ubiquitination of RIP2 in cerebral ischemia. **A**–**C** Western blot analysis of p-NF-κB p65 and IκBα protein levels in WT or OTUD1^−/−^ mice after 24 h MCAO. *n* = 6 mice per group, two-way ANOVA with Tukey’s multiple comparisons test, *P < 0.05 compared with MCAO group of WT mice. **D**–**F** mRNA levels of IL-1β, IL-6 and TNF-α were measured by real-time PCR in brain tissue. Results are representative of three independent experiments, two-way ANOVA with Tukey’s multiple comparisons test, *P < 0.05 compared with MCAO group of WT mice. **G** IP analysis of the endogenous ubiquitination of RIP2 in WT or OTUD1^−/−^ mice with or without the treatment of MCAO

To further decipher the relationship between OTUD1 and RIP2, OTUD1^−/−^ mice were subjected to MCAO. Contrary to the control WT mice, the genetic ablation of OTUD1 significantly increased the neurological deficit scores (Fig. 6A) and the infarct volume (Fig. 6B, C). Moreover, OTUD1 deficiency aggravated neuronal morphological lesion in the hippocampus and cortex visualized by H&E staining (Fig. 6D) and increased the apoptotic cells substantially at 24 h after MCAO (Fig. 6E). To explore the mechanism behind the observed phenotype alteration, NF-κB activity and transcript levels of proinflammatory factors in brain tissues were measured after MCAO. The genetic depletion of OTUD1 evidently induced the activation of NF-κB (Fig. 7A–C) and qRT-PCR acknowledged appreciably boosted IL-1β, IL-6 and TNF-α expression in OTUD1^−/−^ mice (Fig. 7D–F). Meanwhile, ubiquitination of endogenous RIP2 was augmented in OTUD1^−/−^ mice following MCAO (Fig. 7G). Altogether, these findings illustrated that OTUD1 ameliorated brain ischemia by interacting with RIP2, which deubiquitinated of RIP2, and consequently repressed RIP2-induced NF-κB signaling pathway and downstream proinflammatory mediators.

## Discussion

Applying multiple experimental approaches and using both in vitro and in vivo models, our present study showed that protein level of RIP2 and OTUD1 were elevated noticeably in cerebral ischemia induced by MCAO, primary cultured astrocytes and BV2 cells subjected to OGD/R. We also demonstrated that inhibition of RIP2 by GSK559, a small molecule inhibitor, improved the ischemic stroke outcomes, while OTUD1 deficiency exacerbated cerebral ischemic injury. Intriguingly, we provided direct evidence that OTUD1 interacted with RIP2 and cleaved its K63-linked polyubiquitin chains, rather than the K48-linked chains, which led to suppression of the RIP2-induced inflammatory response. These results implied the importance of OTUD1–RIP2 in mediating inflammation in ischemic stroke pathogenesis.

As a serine–threonine–tyrosine kinase, RIP2 is a compulsory downstream signaling molecule of NOD2. Our previous study has reported that NOD2 aggravated cerebral lesion through initiating an inflammatory signaling pathway in mice following cerebral ischemia [7]. However, the role of RIP2 in driven the inflammatory response in ischemic stroke is still unclear. In this study, we first showed that RIP2 protein level was upregulated after brain ischemia and expressed in microglia and astrocytes, indicating RIP2 participated in cerebral ischemic injury. As well-known, the interaction between NOD2 and RIP2 plays a crucial role in maintaining immunity homeostasis and regulating inflammatory response [25]. RIP2 inhibitors targeting the downstream of NOD2 signaling is a potential treatment for inflammatory conditions associated with ischemic stroke. Previous studies suggested small molecule kinase inhibitors of RIP2 presented benefits in animal models of multiple sclerosis [28, 29] and Crohn’s disease-like ileitis [30], positioning RIP2 as a promising target against human inflammatory diseases. In this study, we demonstrated GSK559, an inhibitor of RIP2, produced neuroprotective effects by suppressing inflammatory response after cerebral ischemia. Our results strengthened that RIP2 is a prospective target for regulating inflammatory response in ischemic stroke.

Aside from NOD1/2, other pattern recognition receptors, such as TLRs, also lead to succeeding ubiquitination and activation of RIP2 followed by the activation of NF-κB and MAPK. Downstream of TLR2/3/4, RIP2 is a transducer and integrator of signals for both the innate and adaptive immune systems [31]. Zhang et al. reported the protein level of RIP2 was upregulated in hypoxia and ischemia-induced neuronal cell death by mediating caspase-1 activation, which converted Bid to tBid resulting in caspase-dependent and caspase-independent cell death [32]. Thus, the mechanisms of RIP2 in cerebral ischemia are complicated and required further studies.

OTUD1, a member of the OTU domain family DUBs, suppressed the progression of different tumors [33, 34] and many other diseases through its involvement in the innate immunity response. OTUD1 regulated the production of antiviral cytokines and inflammatory cytokines by MAVS/TRAF3/TRAF6 signaling or NF-κB signaling cascades during viral and fungal infections [18, 35]. OTUD1 also participated in the coordination of intestinal immune responses and autoimmune diseases [17, 36]. However, whether OTUD1 regulates the immuno-inflammatory response in cerebral ischemia has not been studied. The present study illustrated the protein level of OTUD1 was upregulated and localized within microglia and astrocytes both in vivo and in vitro. More importantly, OTUD1 acted as a negative regulator on NF-κB signaling transduction, which directly proved that OTUD1 involved in cerebral ischemia by modulating inflammatory response.

The inflammatory pathway mediated by RIP2 is regulated by ubiquitination associating with many E3 ligases and DUBs [11]. XIAP (X-linked inhibitor of apoptosis), a RING E3 ligase bound to RIP2 of NOD2–RIP2 complex and stimulated RIP2 ubiquitination, which facilitated recruitment of LUBAC for the formation of linear ubiquitin chains and promoted NF-κB activation [37, 38]. The E3 ligase ITCH induced K63-linked polyubiquitination of RIP2 to influence inflammatory signaling pathways [39]. A20 restricted NOD2 induced signals in vitro and in vivo by directly deubiquitination RIP2 [10, 40], while OTULIN inhibited the Met1–Ub formation on RIP2 after NOD2 stimulation to prevent pro-inflammatory signaling [9]. Here, we investigated whether OTUD1 involved in brain ischemia via mediated the deubiquitination of RIP2. RIP2 consists of a kinase domain, an intermediate domain, and a caspase activation and recruitment domain (CARD) [41]. The kinase domain is indispensable for the activation of RIP2 by autophosphorylation and serine–threonine/tyrosine catalytic activity, while the CARD accelerates isostructural bindings with other CARD-containing proteins, such as NOD1 and NOD2 [42]. In cerebral ischemia, we showed OTUD1 interacted with the kinase domain or CARD domain of RIP2, which facilitated OTUD1 to regulate the ubiquitination of RIP2. K48-linked polyubiquitination often induced degradation, whereas K63-linked polyubiquitin chains are known to be involved in protein–protein interactions and mediate signaling pathway stimulation [43]. Strikingly, our findings revealed that OTUD1 interacted with RIP2 through the kinase domain or CARD domain and removed its K63-linked polyubiquitin chains, resulting in the inhibition of RIP2-induced NF-κB activation in cerebral ischemic lesion.

**Fig. 8:**
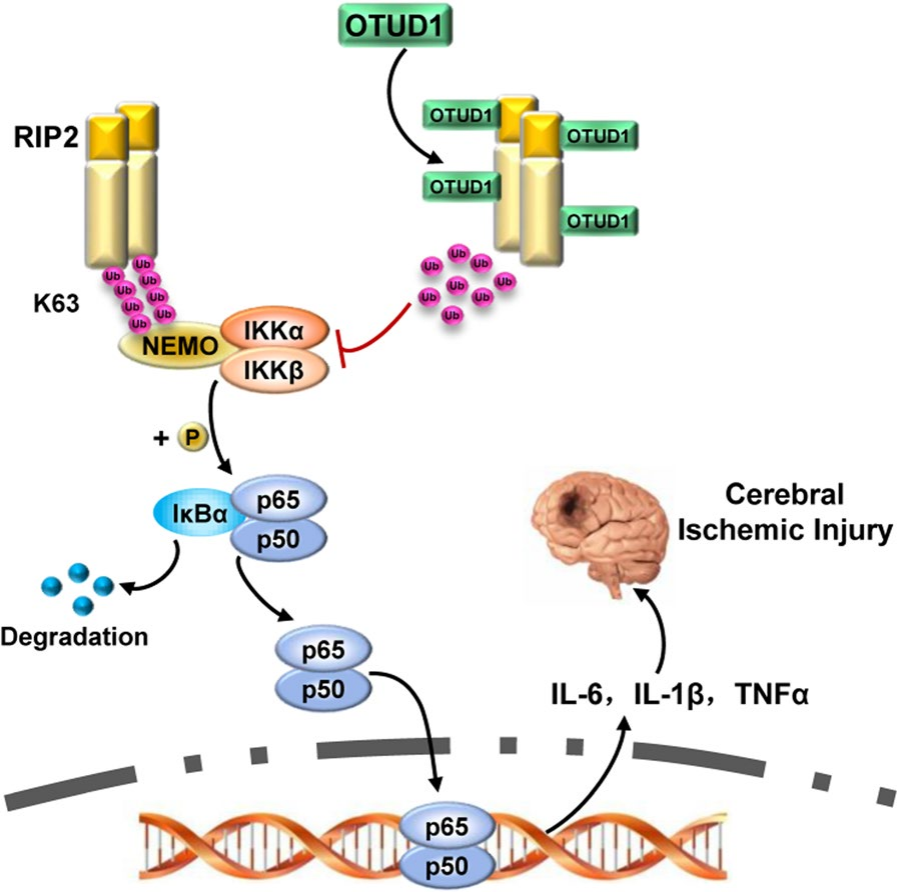
Proposed model of OTUD1 regulated RIP2-mediated inflammation in ischemic stroke. OTUD1 ameliorated brain injury after ischemia through inhibiting RIP2-mediated NF-κB activation to prevent cerebral inflammation by specifically cleaving K63-linked ubiquitination of RIP2

Overall, our study proposed a model for OTUD1–RIP2 interaction and function in cerebral ischemia. Upon stimulation of RIP2, OTUD1 was rapidly recruited to the RIP2 and selectively cut its K63-linked polyubiquitin chains, which alleviated downstream NF-κB-mediated inflammatory response (Fig. 8). Furthermore, we demonstrated OTUD1 deficiency exacerbated brain ischemia and aggravated RIP2-induced inflammatory response in cerebral ischemic injury. Therefore, OTUD1 was a critical regulator that alleviated brain lesion by repressing RIP2-mediated inflammatory reaction in cerebral ischemia.

## Conclusion

Our study revealed RIP2 mediated cerebral ischemic lesion via stimulating inflammatory response both in vivo and in vitro. Meanwhile, inhibition of RIP2 by a small molecule inhibitor GSK559 alleviated the cerebral ischemic outcome. Furthermore, OTUD1 ameliorated brain injury after ischemia through inhibiting RIP2-induced NF-κB signaling pathway to suppress cerebral inflammation by selectively cleaving K63-linked ubiquitination of RIP2. Our results implied a novel ubiquitination regulation mechanism of RIP2 by OTUD1, which in turn could be a potential target for developing novel therapeutical strategy for cerebral ischemia.

### Supplementary Information

**Additional file 1: Figure S1.** OTUD1 had no effect on RIP2 protein degradation in BV2 cells. (A) Western blot analysis of RIP2 protein levels after OTUD1 overexpression in in BV2 cells transfected with plasmids expressing Flag-OTUD1. (B) Western blot analysis of RIP2 in BV2 cells transfected with siRNA OTUD1.

## Data Availability

All data generated in this study are included in this manuscript.

## Abbreviations

CARD: Caspase activation and recruitment domain
DUB: Deubiquitinating enzymes
DAMPs: Damage-associated molecular patterns
MCAO: Middle cerebral artery occlusion
NOD: Nucleotide-binding oligomerization domain
OTU: Ovarian tumor protease
OGD/R: Oxygen glucose deprivation and reoxygenation
RIP2: Receptor-interacting kinase 2
Ub: Ubiquitination
WT: Wild type
