# Supplementary figures and images for: OTUD1 ameliorates cerebral ischemic injury through inhibiting inflammation by disrupting K63-linked deubiquitination of RIP2
Source: J Neuroinflammation. 2023 Nov 27;20:281. doi: 10.1186/s12974-023-02968-7 (PMC10680203; doi:10.1186/s12974-023-02968-7)

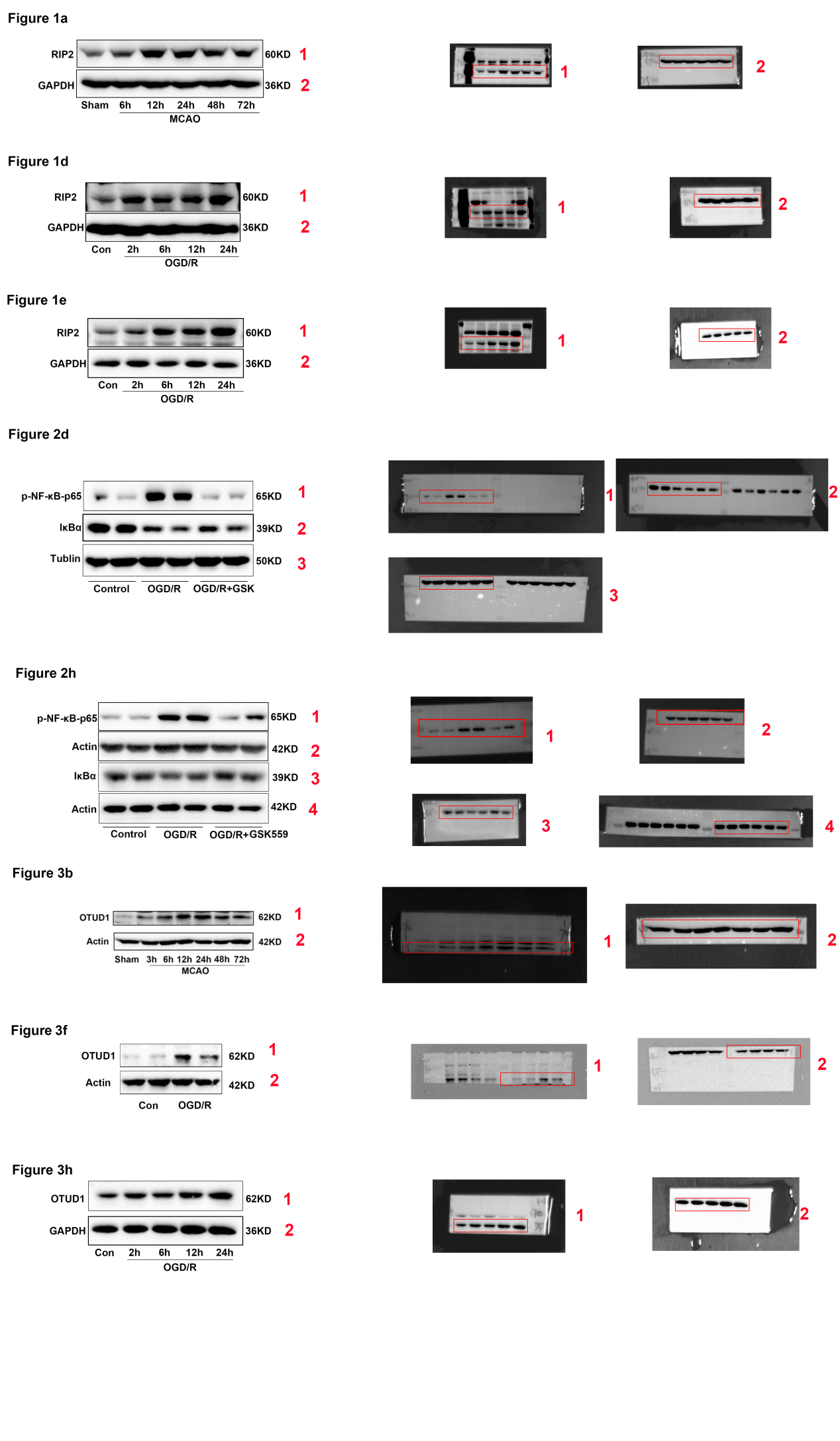

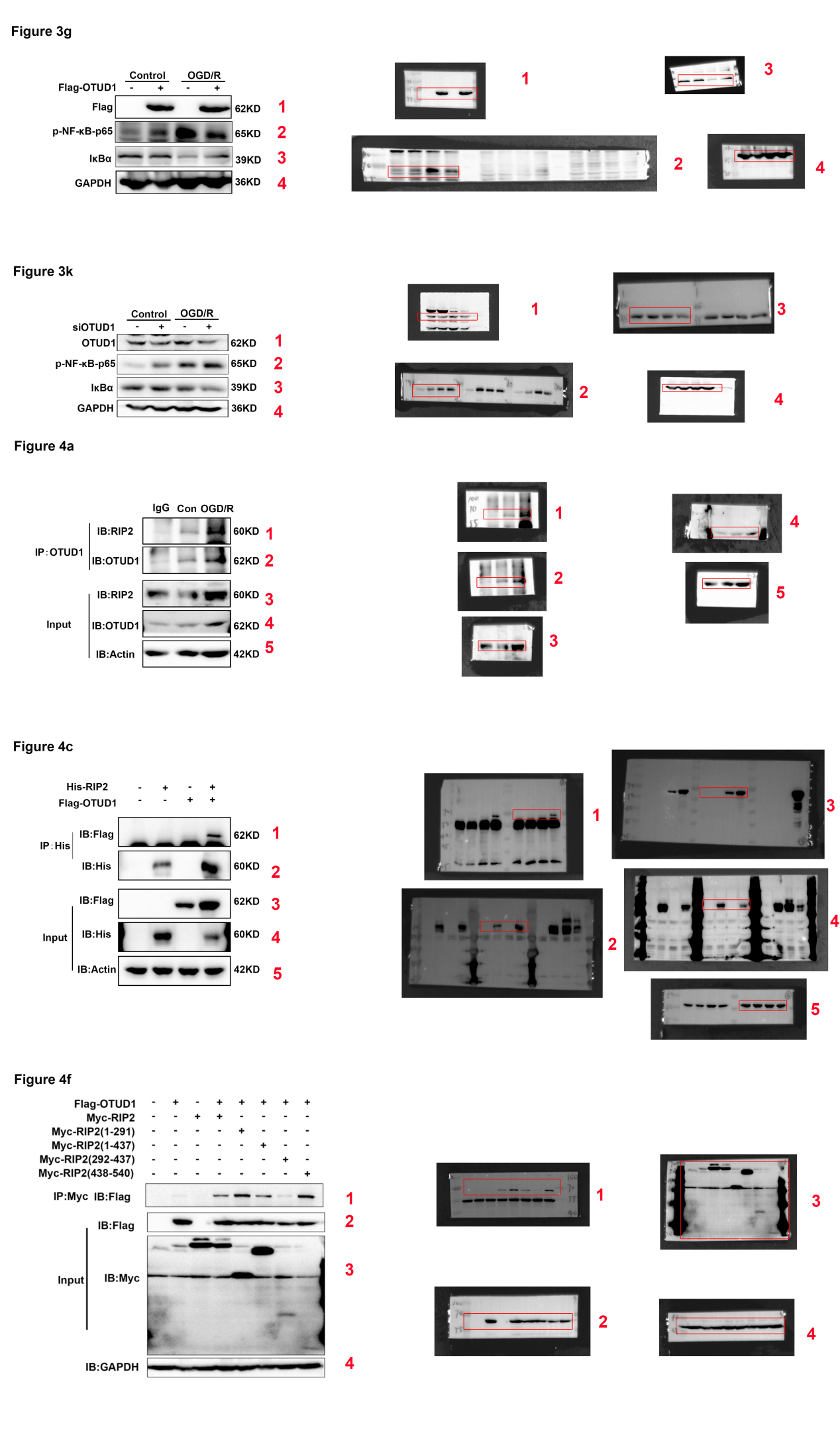

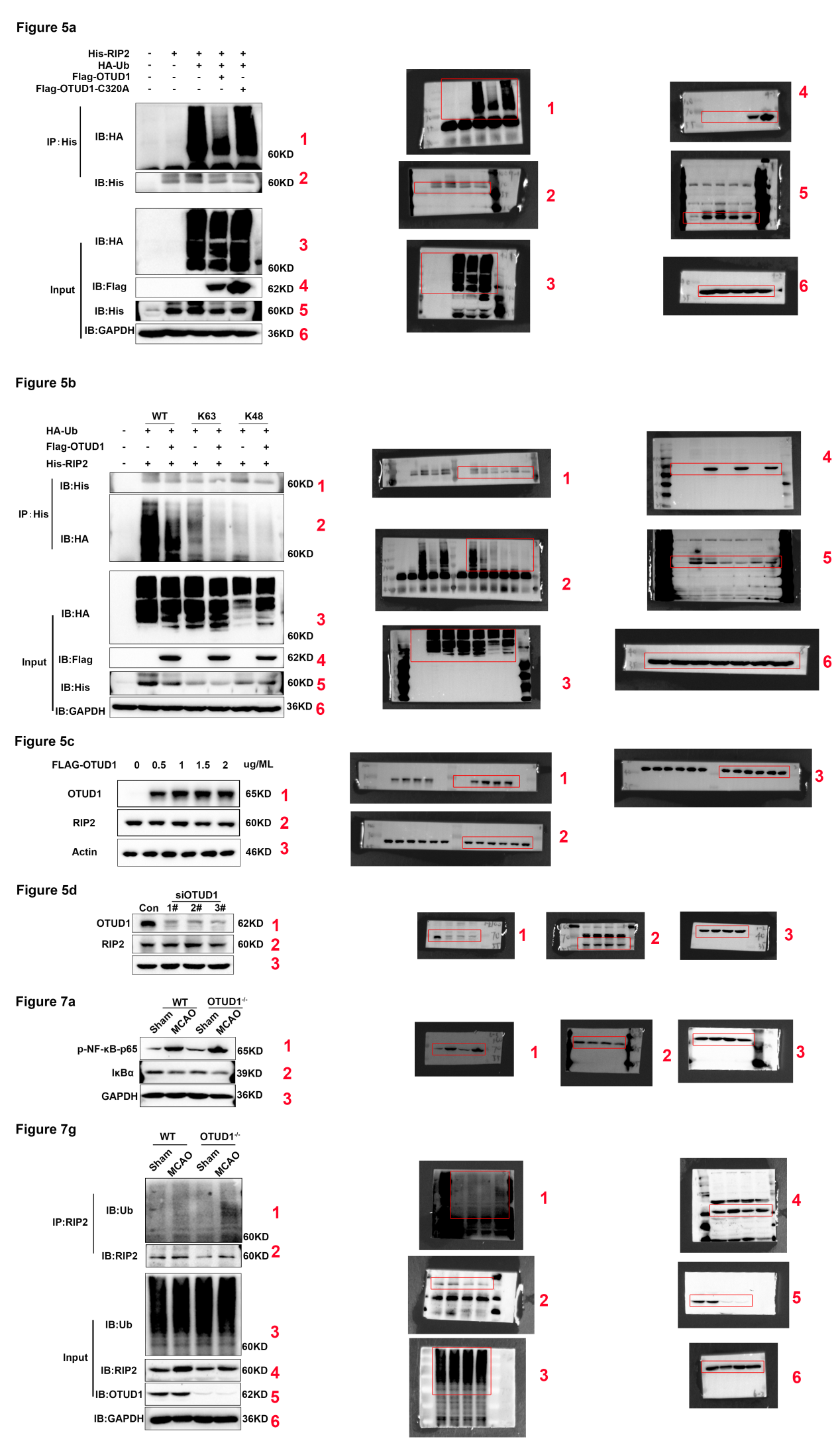


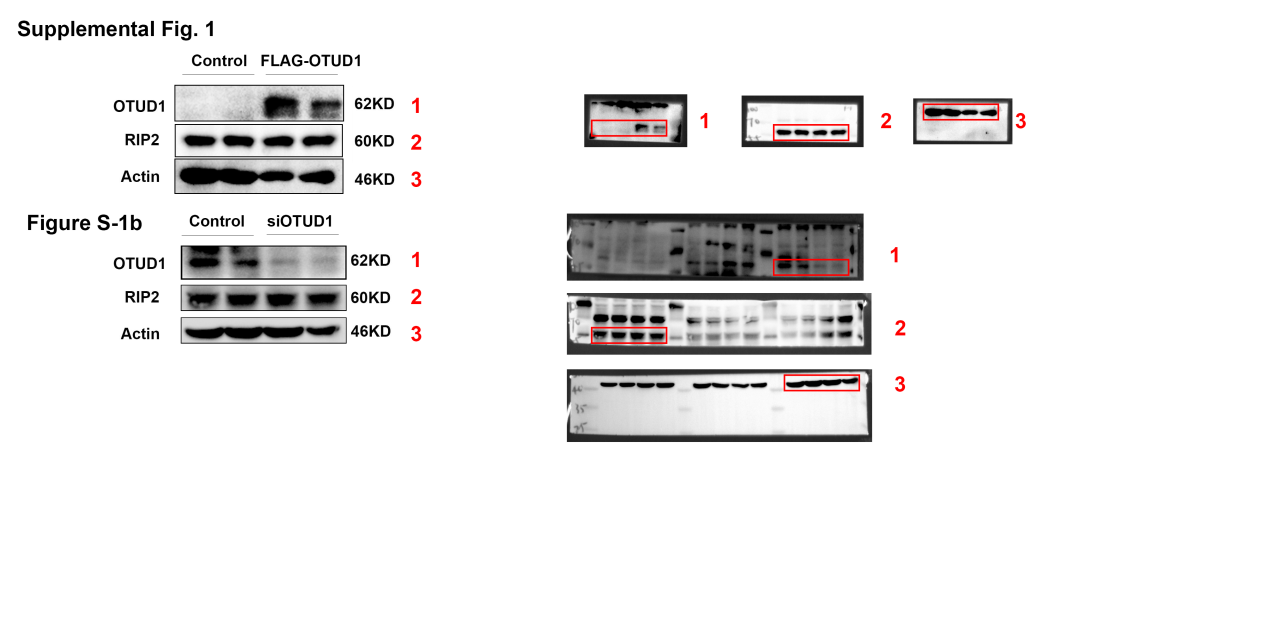

Supplement: Supplementary file 1 — Additional file 1: Figure S1. OTUD1 had no effect on RIP2 protein degradation in BV2 cells. (A) Western blot analysis of RIP2 protein levels after OTUD1 overexpression in in BV2 cells transfected with plasmids expressing Flag-OTUD1. (B) Western blot analysis of RIP2 in BV2 cells transfected with siRNA OTUD1. [file 12974_2023_2968_MOESM1_ESM.docx]
